# Reduced Lymphatic Reserve in Heart Failure With Preserved Ejection Fraction

**DOI:** 10.1016/j.jacc.2020.10.022

**Published:** 2020-12-15

**Authors:** Giacomo Rossitto, Sheon Mary, Christine McAllister, Karla Bianca Neves, Laura Haddow, John Paul Rocchiccioli, Ninian Nicholas Lang, Clare Louise Murphy, Rhian Merry Touyz, Mark Colquhoun Petrie, Christian Delles

**Affiliations:** a Institute of Cardiovascular and Medical Sciences, University of Glasgow, Glasgow, United Kingdom; b Department of Medicine, Università degli Studi di Padova, Padova, Italy; c Clinical Research Facility, Queen Elizabeth University Hospital, Glasgow, United Kingdom; d Department of Cardiology, Golden Jubilee National Hospital, Clydebank, United Kingdom; e Department of Cardiology, Royal Alexandra Hospital, Paisley, United Kingdom

**Keywords:** edema, interstitium, heart failure, lymphatic, microcirculation, preserved ejection fraction, vascular rarefaction, BNP, B-type natriuretic peptide, BP, blood pressure, CVP, central venous pressure, DD, deep dermis, ESD, epidermis and superficial dermis, HC, healthy control, HF, heart failure, HFpEF, heart failure with preserved ejection fraction, K_f_, microvascular filtration coefficient, P_i_, isovolumetric pressure, P_V_, venous pressure, VEGF, vascular endothelial growth factor

## Abstract

**Background:**

Microvascular dysfunction plays an important role in the pathogenesis of heart failure with preserved ejection fraction (HFpEF). However, no mechanistic link between systemic microvasculature and congestion, a central feature of the syndrome, has yet been investigated.

**Objectives:**

This study aimed to investigate capillary–interstitium fluid exchange in HFpEF, including lymphatic drainage and the potential osmotic forces exerted by any hypertonic tissue Na^+^ excess.

**Methods:**

Patients with HFpEF and healthy control subjects of similar age and sex distributions (n = 16 per group) underwent: 1) a skin biopsy for vascular immunohistochemistry, gene expression, and chemical (water, Na^+^, and K^+^) analyses; and 2) venous occlusion plethysmography to assess peripheral microvascular filtration coefficient (measuring capillary fluid extravasation) and isovolumetric pressure (above which lymphatic drainage cannot compensate for fluid extravasation).

**Results:**

Skin biopsies in patients with HFpEF showed rarefaction of small blood and lymphatic vessels (p = 0.003 and p = 0.012, respectively); residual skin lymphatics showed a larger diameter (p = 0.007) and lower expression of lymphatic differentiation and function markers (LYVE-1 [lymphatic vessel endothelial hyaluronan receptor 1]: p < 0.05; PROX-1 [prospero homeobox protein 1]: p < 0.001) compared with control subjects. In patients with HFpEF, microvascular filtration coefficient was lower (calf: 3.30 [interquartile range (IQR): 2.33 to 3.88] l × 100 ml of tissue^–1^ × min^–1^ × mm Hg^–1^ vs. 4.66 [IQR: 3.70 to 6.15] μl × 100 ml of tissue^–1^ × min^–1^ × mm Hg^–1^; p < 0.01; forearm: 5.16 [IQR: 3.86 to 5.43] l × 100 ml of tissue^–1^ × min^–1^ × mm Hg^–1^ vs. 5.66 [IQR: 4.69 to 8.38] μl × 100 ml of tissue^–1^ × min^–1^ × mm Hg^–1^; p > 0.05), in keeping with blood vascular rarefaction and the lack of any observed hypertonic skin Na^+^ excess, but the lymphatic drainage was impaired (isovolumetric pressure in patients with HFpEF vs. control subjects: calf 16 ± 4 mm Hg vs. 22 ± 4 mm Hg; p < 0.005; forearm 17 ± 4 mm Hg vs. 25 ± 5 mm Hg; p < 0.001).

**Conclusions:**

Peripheral lymphatic vessels in patients with HFpEF exhibit structural and molecular alterations and cannot effectively compensate for fluid extravasation and interstitial accumulation by commensurate drainage. Reduced lymphatic reserve may represent a novel therapeutic target.

Heart failure (HF) is a leading cause of morbidity and mortality (1). Current trends of increased HF hospitalizations are mostly driven by HF with preserved ejection fraction (HFpEF), carrying a 1-year prognosis almost as poor as in patients with HF with reduced ejection fraction (2, 3, 4).

HFpEF is a clinical syndrome closely associated with multiple cardiovascular comorbidities and risk factors, such as hypertension, obesity, coronary artery disease, diabetes mellitus, atrial fibrillation, and chronic kidney disease (5). The notion that these comorbidities not only are associated with HFpEF, but also may be directly involved in its pathogenesis via comorbidity-associated inflammation and coronary microvascular dysfunction (6) has gained support from a large body of postmortem, noninvasive, and invasive evidence (7, 8, 9). In addition to the coronary vascular bed, peripheral vessels also appear dysfunctional (8). In particular, impaired systemic vasodilator reserve and low skeletal muscle capillary density, as well as low peripheral O_2_ extraction paralleling microvascular rarefaction, were reported as determinants of exercise intolerance in patients with HFpEF (4,10, 11, 12). Such findings challenged the paradigm of a purely cardiac disorder in favor of a more systemic phenomenon (13). However, whether and how a dysfunctional microcirculation could directly impact congestion, “the core of the HF syndrome” (5), has not yet been investigated.

In recent years, understanding of body fluid homeostasis has evolved through reappraisal of tissue sodium (Na^+^) accumulation. This phenomenon was proposed to be water-independent (14) and to induce a hypertonicity-driven lymphatic network expansion in order to facilitate local Na^+^ drainage (15). Notably, tissue Na^+^ excess was found in most of the conditions and risk factors associated with the clinical HFpEF syndrome (i.e., older age, hypertension, diabetes, and chronic kidney disease) (16, 17, 18), but its hypertonic nature and functional relevance to fluid homeostasis in HFpEF lacks demonstration.

Therefore, the HAPPIFY (Heart fAilure with Preserved ejection fraction: Plethysmography for Interstitial Function and skin biopsY) study was designed to investigate capillary–interstitium fluid exchange and to test the hypothesis that an osmotic effect secondary to high interstitial Na^+^ levels could impact microvessels and result in excess fluid extravasation and edema in patients with HFpEF.

## Methods

### Study protocol and subjects

Subjects with stable HFpEF, identified from outpatient HF clinics in Glasgow, United Kingdom (n = 16), and volunteers of similar age and sex distributions, with no history of cardiovascular or renal disease, hypertension, or diabetes (healthy control [HC] subjects; n = 16), were recruited between August 2017 and December 2018. HFpEF diagnosis was per the 2016 European Society of Cardiology guideline definition: 1) signs or symptoms of HF; 2) elevated B-type natriuretic peptide (BNP) >35 pg/ml; 3) ejection fraction ≥50%; and 4) evidence of structural heart disease (left atrial enlargement or left ventricular hypertrophy) or diastolic dysfunction (19). Exclusion criteria were a history of recent (<3 months) cerebrovascular event, myocardial infarction, or coronary revascularization; significant valve disease; unstable coronary artery disease; hypertrophic or infiltrative cardiomyopathy or constrictive pericarditis; chronic kidney disease stage >3; idiopathic edema or capillary leak syndrome, myxedema, or lymphatic obstruction; systemic inflammation at the time of study visit; active malignancy; any major hypercoagulable state or history of venous thrombosis or embolism on no ongoing anticoagulation; and incapacity.

Eligibility and main visits were conducted in the morning, in a temperature-controlled room. Subjects did not consume caffeine, alcohol, or food; did not smoke; and avoided vasoactive or diuretic medications for >4 h prior to assessments. The study procedures included history and physical examination; full blood count, BNP, hemoglobin A1c, and renal, liver, and thyroid function; urinary albumin-to creatinine ratio (morning spot sample); simultaneous bilateral brachial and calf blood pressure (BP) measurement; pulsed wave velocity; echocardiography; flow-mediated dilatation; venous occlusion strain gauge plethysmography; and a gluteal skin punch biopsy. Participants who were not on anticoagulants and who had no history of severe obstructive iliac artery disease were offered to participate in an optional substudy, involving a larger surgical excision of skin and subcutaneous fat tissue around the site of the punch biopsy, for dissection of resistance arteries and molecular biology. This study conforms to the Declaration of Helsinki and the protocol was approved by the West of Scotland Research Ethics Committee 3 (ref. 17/WS/0091) and Greater Glasgow and Clyde NHS Research and Development (ref. GN17CA152).

### Tissue samples

All participants (except for 1 patient, owing to ineffective anesthesia) underwent a 4-mm skin punch biopsy on a gluteal external upper quadrant, after topical numbing with Na^+^-free lidocaine cream (LMX4, Ferndale Pharma Group, Ferndale, Michigan) as described (20). One-half of the skin sample was fixed in paraformaldehyde 2% for histology, and the other one-half was immediately frozen and stored at –80°C until chemical analysis.

A skin portion of the larger biopsies obtained from participants eligible and consenting also to the substudy was immediately frozen for molecular biology (7 patients with HFpEF and 10 HC subjects); the remaining tissue was used to dissect small resistance arteries for ex vivo functional testing of endothelium-dependent and endothelium-independent relaxation by wire myography.

### Skin histochemical analysis

As reported (20), frozen skin samples were cut into a superficial layer (including the epidermis and superficial dermis [ESD]) and deep dermis (DD) layer in a cold room, to prevent evaporation. Tissue water content was estimated as wet weight − dry weight (DW), determined after desiccation at 65°C for >40 h to stable weight. Tissue Na^+^ and K^+^ were measured in the HNO_3_-digested dry samples by flame photometry (410C, Sherwood Scientific, Cambridge, United Kingdom) and expressed as absolute content (mmol/g DW) or concentration (mmol/l, after normalization by tissue water).

### Skin microvascular imaging

Paraffin-embedded skin sections (5-μm thick) including both epidermis and dermis were stained with anti-human LYVE-1 (lymphatic vessel endothelial hyaluronan receptor 1) antibody (R&D Systems, Minneapolis, Minnesota; 3 μg/ml, overnight, 4°C; matched isotype goat immunoglobulin G as negative control), after overnight low-temperature antigen retrieval (Unitrieve; Innovex Biosciences, Richmond, California) and nonspecific signal blocking. Alexa fluor 488–conjugated secondary antibody (2 μg/ml; Invitrogen, Carlsbad, California) and Ulex europaeus agglutinin I lectin (10 μg/ml; Vector Laboratories, Burlingame, California) were used to stain LYVE-1^+^ lymphatic vessels and blood endothelial cells, respectively. Tiled images of the entire epidermal line and the subpapillary dermis were acquired at 20× magnification. Blinded automated image analysis for microvessels quantification in the 600-μm-thick dermal area starting from the epidermis–dermis junction and spanning the entire length of the biopsy was performed with ImageJ version 1.52q (National Institutes of Health, Bethesda, Maryland), by pre-defined homogeneous thresholding criteria and the “analyze particles” function (Supplemental Figure 1).

### Skin gene expression analysis

RNA was extracted from gluteal surgical biopsies. After preamplification (TaqMan PreAmp technology, Applied Biosystems, Foster City, California) of target complementary DNA, a Custom TaqMan Array Card (Thermo Fisher Scientific, Waltham, Massachusetts) was used to perform quantitative gene expression of markers or growth and transcription factors specific to blood or lymphatic vessels. Gene expression levels were compared and presented as ΔCt values, with β-actin as housekeeping gene.

### Plethysmography and microvascular fluid dynamics

Strain gauge plethysmography (EC6, Hokanson, Carmel, Indiana), measuring limb volume changes as ml/100 ml of tissue, was used to assess forearm and calf arterial blood flow, peripheral venous pressure (P_V_) (as a surrogate for central venous pressure [CVP]) and net fluid extravasation toward the interstitium at increasing venous occluding pressures, as described previously (21, 22, 23, 24, 25).

Briefly, strain gauges were positioned on the nondominant forearm and on the ipsilateral calf and were maintained at the height of the right atrium. Inflatable cuffs for venous occlusion were placed proximal to the strain gauges. P_V_ was determined by gradually increasing the occlusion pressure, sustained until any limb volume change was detected. Arterial blood flow was determined from the rate of change in limb volume after consecutive cycles of sudden venous occlusion to 45 mm Hg (E20 Rapid Cuff Inflator, D.E. Hokanson Inc., Bellevue, Washington), as described previously (22). Arterial resistance to flow was calculated as: (mean BP − P_V_) / blood flow (24).

To assess microvascular filtration parameters, we used cumulative 8-mm Hg occluding pressure steps, lasting 3.5 min each and starting at the first multiple exceeding P_V_, up to a maximum of 56 mm Hg or less if diastolic BP was lower. The pressure applied to the cuff and transmitted to the veins induces their distension and filling, with a curvilinear initial phase and a later plateau (Supplemental Figure 2A). However, above a certain equilibrium pressure up to which any fluid filtering across the microvascular interface is being removed at an equivalent rate by lymphatic drainage, interstitial fluid accumulation occurs, thus resulting in a linear increase in limb volume (Supplemental Figure 2B). This pressure threshold is called isovolumetric pressure (P_i_). The time course (i.e., slope) of this interstitial fluid accumulation was calculated as the averaged first derivative of the plethysmographic tracing for each capillary hydrostatic pressure (= pressure applied to the cuff) step, in portions devoid of motion artifacts and excluding the venous filling phase (LabChart, ADInstruments, Sydney, Australia); these slopes are proportional to hydrostatic pressure by a coefficient called microvascular filtration coefficient (K_f_) (μl × 100 ml of tissue^–1^ × min^–1^ × mm Hg^–1^) (Supplemental Figure 2C) (21). The filtration coefficient, as well as the pressure when extravasation and lymphatic drainage balance and the net increase in tissue volume is null (i.e., the intercept of the linear association with the x-axis; P_i_), were determined for each limb and participant, blindly to group allocation, by least-squares regression (Prism, version 8; GraphPad Software, San Diego, California). Limbs with <3 points free of motion artifacts for regression fitting (21,26) or with pre-defined limb-specific exclusion criteria were excluded from analysis.

### Statistical analysis

Recruitment was stopped after 16 of 20 initially estimated subjects per group, when interim analysis showed futility in relation to the primary hypothesis of a higher K_f_ in patients with HFpEF compared with HC subjects. All statistical analyses were performed using Prism and SPSS version 25 (IBM Corporation, Armonk, New York). Categorical variables were compared by chi-square test. Continuous variables were tested for normality of distribution by graphical plotting and Kolmogorov-Smirnov test. Parametric or nonparametric unpaired unadjusted tests were used for comparison of primary (microvascular) and secondary endpoints between groups, accordingly. Least-squares fit was used for both nonlinear regression and linear regression. The α level was set at 0.05, and all statistical tests were 2-tailed.

An extended description of the study methods is available in the Supplemental Appendix.

## Results

### Subject characteristics and vascular function

Clinical characteristics of the 2 study groups are provided in Table 1. Patients with HFpEF and HC subjects were similar for age and sex distributions. Patients with HFpEF showed typical characteristics, with high prevalence of hypertension, obesity, paroxysmal or permanent atrial fibrillation, left ventricular hypertrophy and signs of atrial remodeling or diastolic dysfunction (Supplemental Table 1), diabetes, and chronic kidney disease. Compared with HC subjects, they had higher BNP, albuminuria, and plasma urea, and lower eGFR and hematocrit, but similar plasma albumin.

**Table 1:** Subject Characteristics

	HC Subjects (n = 16)	Patients With HFpEF (n = 16)	p Value
Female	11 (69)	10 (63)	0.710
Age, yrs	68 ± 5	72 ± 6	0.060
BMI, kg/m^2^	25.1 ± 2.9	33.9 ± 4.4	<0.001
Overweight/obese	8 (50)/1 (6)	4 (25)/12 (75)	<0.001
BSA, m^2^	1.78 ± 0.21	2.00 ± 0.28	0.021
SBP, mm Hg	130 ± 14	146 ± 21	0.017
DBP, mm Hg	73 ± 8	71 ± 14	0.659
HR, beats/min	60 ± 7	65 ± 16	0.256
Left atrial volume index, ml/m^2^	23.3 ± 6.1	46.8 ± 12.8	<0.001
Left ventricular mass index, g/m^2^	84.3 ± 17.9	127.8 ± 26.6	<0.001
Left ventricular ejection fraction, %	63.2 ± 3.5	60.8 ± 6.7	0.222
E/e′	8.0 ± 1.6	10.8 ± 2.9	0.001
Comorbidity			
Hypertension	—	15 (94)	<0.001
Coronary artery disease	—	3 (20)	0.060
Atrial fibrillation	—	11 (69)	<0.001
Diabetes mellitus	—	7 (44)	0.003
Chronic kidney disease	—	7 (44)	0.003
Smoking (current/prior)	0 (0)/2 (13)	3 (19)/7 (44)	0.011
Medication			
ACE inhibitor/ARB	—	14 (88)	<0.001
BB	—	14 (88)	<0.001
CCB	—	3 (19)	0.069
Diuretic (loop/thiazide)	—	15 (94)/1 (6)	<0.001
MRA	—	1 (6)	0.310
Long-acting nitrates	—	2 (13)	0.144
Statin	—	8 (50)	0.001
Digoxin	—	5 (31)	0.015
Aspirin	—	4 (25)	0.033
Anticoagulation	—	7 (44)	0.003
Na^+^	140 (139–142)	140 (136–142)	0.361
K^+^	4.5 (4.2–4.7)	4.2 (4.0–4.7)	0.224
Urea, mmol/l	5.5 (4.7–6.0)	6.4 (5.9–9.8)	0.007
Creatinine, μmol/l	71 (59–84)	79 (67–90)	0.138
eGFR (CKD-EPI), ml/min/1.73 m^2^	81.7 ± 11.0	70.9 ± 17.0	0.042
Urinary ACR, mg/gCr	4.8 (3.2–6.1)	33.9 (10.7–87.4)	0.001
Hemoglobin, g/l	145 ± 8.8	133 ± 17	0.022
Hematocrit, %	44.1 ± 2.5	40.5 ± 4.7	0.013
Albumin, g/l	39.3 ± 3.1	37.8 ± 2.7	0.155
BNP, pg/ml	24 (16–32)	170 (93–320)	<0.001

Values are n (%), mean ± SD, or median (interquartile range). Chronic kidney disease was defined as eGFR <60 ml/min/1.73 m^2^.

ACE = angiotensin-converting enzyme; ACR = albumin to creatinine ratio; ARB = angiotensin receptor blocker; BB = beta-blocker; BMI = body mass index; BNP = B-type natriuretic peptide; BSA = body surface area (Mosteller); CCB = calcium-channel blocker; CKD-EPI = Chronic Kidney Disease Epidemiology Collaboration; DBP = diastolic blood pressure; E/e′ = average of septal and lateral velocity ratio; eGFR = estimated glomerular filtration rate; HC = healthy control; HFpEF = heart failure with preserved ejection fraction; HR = heart rate; MRA = mineralocorticoid receptor antagonist; SBP = systolic blood pressure.

Classical assessment of peripheral vascular function also revealed higher carotid-femoral pulsed wave velocity, as a measure of stiffness of large arteries, and lower brachial flow-mediated dilatation, which was accompanied by lower post-ischemic reactive hyperemia (Supplemental Table 2, Supplemental Figure 3). Consistently, ex vivo vasodilation of subcutaneous resistance arteries was reduced in patients with HFpEF compared with HC subjects (Supplemental Figure 4).

### Skin salt

**Figure 1:**
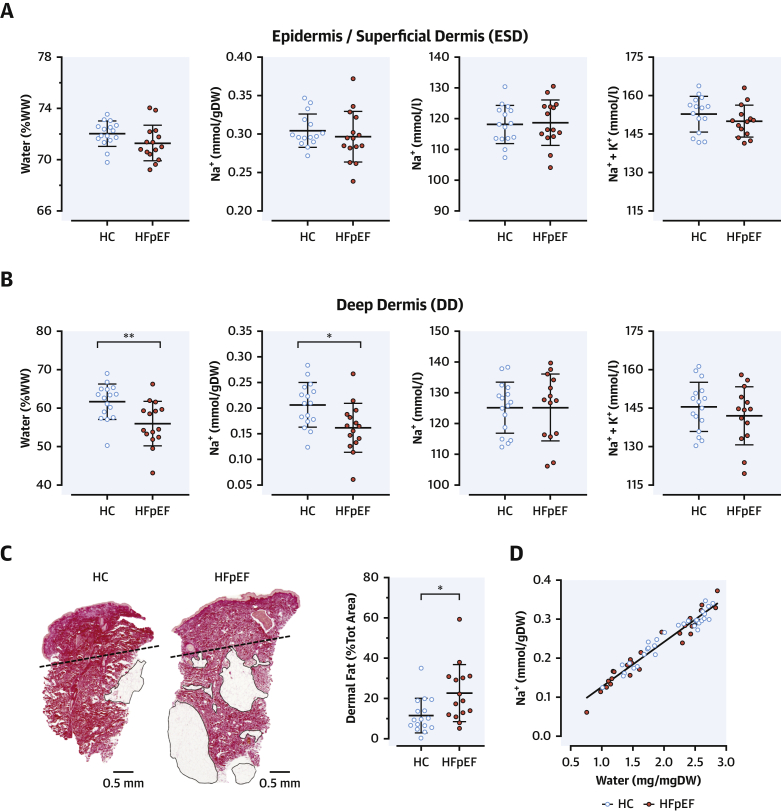
Chemical Analysis of the Skin **(A, B)** Water content (% of wet weight [WW]), Na^+^ content (mmol/g of tissue dry weight [DW]), Na^+^ concentration, and [Na^+^ + K^+^] concentration (mmol/l of tissue water) in the outer (epidermis and superficial dermis [ESD]) and the inner (deep dermis [DD]) layers of the skin biopsies. **(C)** Representative skin sections from a healthy control (HC) subject and a patient with heart failure with preserved ejection fraction (HFpEF) (picrosirius red staining). The **dashed line** identifies the cutting plane for separation of ESD and DD in all study samples, scale bars are at the bottom, and **black-circled areas** represent dermal fat, quantified in the sections as % of biopsy area and were more abundant in patients with HFpEF than in HC subjects. **(B)** Excess dermal fat parallels the reduced volume of distribution of water and Na^+^ and, accordingly, their reduced content observed in the DD of patients with HFpEF. All data in panels **A–C** are presented as mean ± SD; ∗ p < 0.05, ∗∗p < 0.01. **(D)** Association between Na and water content, including both ESD and DD values; the slope of the regression line was the same between study groups.

The chemical analysis of gluteal skin biopsies revealed that water content (% wet weight [mg/mg dry weight] [not shown]), Na^+^ concentration (mmol/l of water), and total Na^+^ content (mmol/g DW) in the ESD were similar between patients with HFpEF and HC subjects (Figure 1A). In the DD, water and Na^+^ content were lower in patients with HFpEF than in HC subjects (Figure 1B), in keeping with the distribution of body mass index and dermal fat (Figure 1C, Supplemental Figure 5), which limits the volume of distribution of both water and electrolytes in the tissue while leaving their relative representation overall unaffected. DD Na^+^ and (Na^+^ + K^+^) concentrations (i.e., upon content normalization for water) were similar between groups, as in ESD. Na^+^ + K^+^ concentration consistently fell within a physiological range (140 to 155 mmol/l) (20) in both layers. Likewise, the slope of the regression line for skin water and Na^+^ content did not differ by group (p = 0.810) (Figure 1D) or tissue layer (ESD vs. DD, p = 0.922) (not shown). In summary, we did not detect any hypertonic, water-independent accumulation of Na^+^ in the skin of patients with HFpEF.

### Rarefaction of skin blood and lymphatic microvessels and differential gene expression

#### Blood microvessels

**Figure 2:**
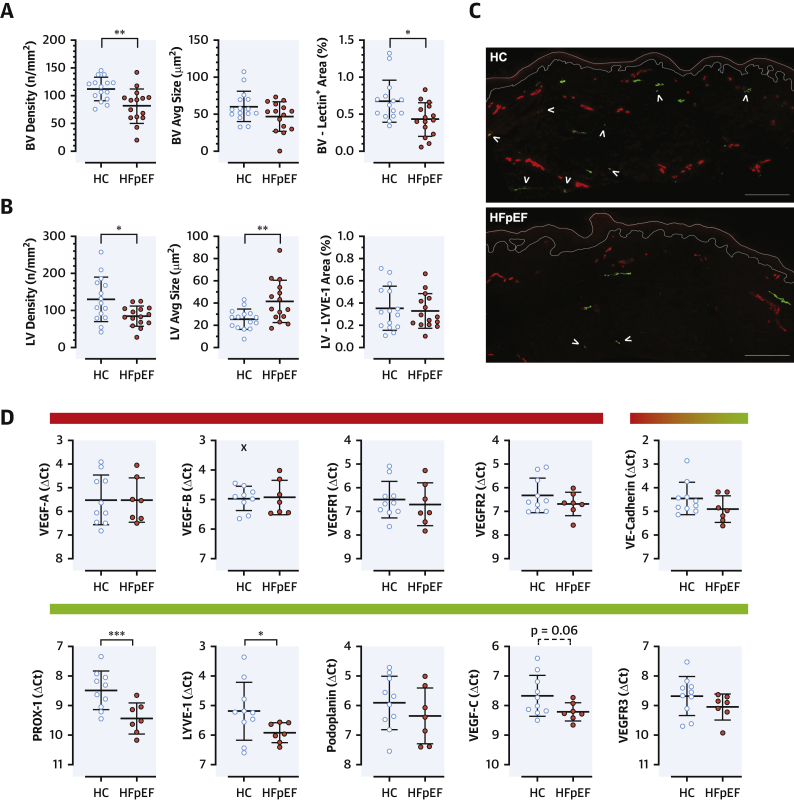
Skin Microvascular Anatomy and Gene Expression Skin microvascular anatomy assessed as density (number of vessels/mm^2^ tissue), average cross-sectional area of the vessels (average size; μm^2^), and total stain^+^ area (expressed as percentage of the dermal area) of the vessels identified in the section. **(A)** Blood vessels (BVs) showed a reduction in number and total area^+^; **(B)** lymphatic vessels (LVs) were reduced in number but, on average, were larger in size in patients with HFpEF compared with HC subjects. **(C)** Representative sections, showing blood vessels (lectin^*+*^**[red]**) and lymphatic vessels (LYVE-1 [lymphatic vessel endothelial hyaluronan receptor 1] **[green]**); **arrowheads** indicated small terminal lymphatic vessels, reduced in HFpEF compared with HC subjects, and larger vessels appeared unaffected. Scale bar at bottom right = 200 μm. **(D)** Gene expressions, presented as ΔCt (the higher the value, the lower the gene expression; β-actin as housekeeping gene); **colored bars** on top to indicate blood **(red)**, lymphatic **(green)**, or mixed vascular specificity; x = automatically detected outlier (ROUT = 1%) (GraphPad Prism). All data are presented as mean ± SD; ∗p < 0.05, ∗∗p < 0.01, ∗∗∗p < 0.001. PROX-1 = prospero homeobox protein 1; VEGF = vascular endothelial growth factor; VEGFR = vascular endothelial growth factor receptor; other abbreviations as in Figure 1.

Patients with HFpEF had dermal rarefaction of blood microvessels compared with HC subjects (p = 0.003) (Figures 2A and 2C), independent of age (Spearman correlation with number of vessels: r = –0.141, p = 0.449). No significant difference was observed in the size of skin vessels or in the gene expression of vascular endothelial growth factor A (VEGF-A) and VEGF-B and their blood-vessel specific receptors (VEGF receptor 1 [VEGFR1] and VEGFR2); the expression of vascular endothelial cadherin, present in both blood and lymphatic vessels, was not different either (Figure 2D).

#### Lymphatic microvessels

Lymphatic vessels were similarly reduced in number (p = 0.012) but were larger (p = 0.007) in patients with HFpEF compared with HC subjects (Figures 2B and 2C). Neither number nor average vascular size showed significant associations with age (r = –0.009, p = 0.963; and r = 0.307, p = 0.099, respectively). In patients with HFpEF, skin PROX-1 (prospero homeobox protein 1, a LEC-specific transcription factor driving development and dynamic maintenance of lymphatic vessels and valves) (27,28) and LYVE-1 (a marker of lymphatic endothelial cells expressed since the most initial vessels), but not podoplanin (a glycoprotein generally expressed in the endothelium of lymphatic vessels above a certain diameter) (29), were reduced compared with HC subjects. VEGF-C showed a similar trend (p = 0.06), but the expression of its lymphatic-specific receptor VEGFR3 did not differ between groups (Figure 2D). Overall, both structural and molecular skin lymphatic alterations were detected in patients with HFpEF.

### Microvascular fluid dynamics: Microfiltration and lymphatic clearance

Patients with HFpEF had forearm and calf mean blood pressure, rest limb blood flow (normalized for milliliters of tissue), and arterial resistance similar to HC subjects; at variance, their rest P_V_ was higher, although not to values suggestive of overt decompensation (Table 2).

**Table 2:** Microvascular Dynamics

	HC Subjects (n = 16)	Patients With HFpEF (n = 16)	p Value
Forearm			
Mean arterial pressure, mm Hg	92 ± 7	94 ± 12	0.529
Blood flow, ml × 100 ml of tissue^–1^ × min^–1^	1.9 ± 0.8	1.9 ± 0.9	0.992
Arterial peripheral resistance, mm Hg/ml × 100 ml tissue^–1^ × min^–1^	50.1 ± 14.5	56.3 ± 29.5	0.459
Venous pressure, mm Hg	5 (4–6)	8 (7–10)	<0.001
Filtration coefficient (K_f_), μl × 100 ml of tissue^–1^ × min^–1^ × mm Hg^–1^	5.66 (4.69–8.38)	5.16 (3.86–5.43)	0.234
Isovolumetric pressure, mm Hg	25 ± 5	17 ± 4	<0.001
Calf			
Mean arterial pressure, mm Hg	94 ± 8	96 ± 14	0.555
Blood flow, ml × 100 ml of tissue^–1^ × min^–1^	3.3 ± 1.6	3.6 ± 1.4	0.573
Arterial peripheral resistance, mm Hg/ml × 100 ml tissue^–1^ × min^–1^	33.1 ± 15.4	29.3 ± 11.8	0.459
Venous pressure, mm Hg	5 (4–5)	7 (7–9)	<0.001
Filtration coefficient (K_f_) μl × 100 ml of tissue^–1^ × min^–1^ × mm Hg^–1^	4.66 (3.70–6.15)	3.30 (2.33–3.88)	0.008
Isovolumetric pressure, mm Hg	22 ± 4	16 ± 4	0.003

Values are mean ± SD or median (interquartile range).

Abbreviations as in Table 1.

**Figure 3:**
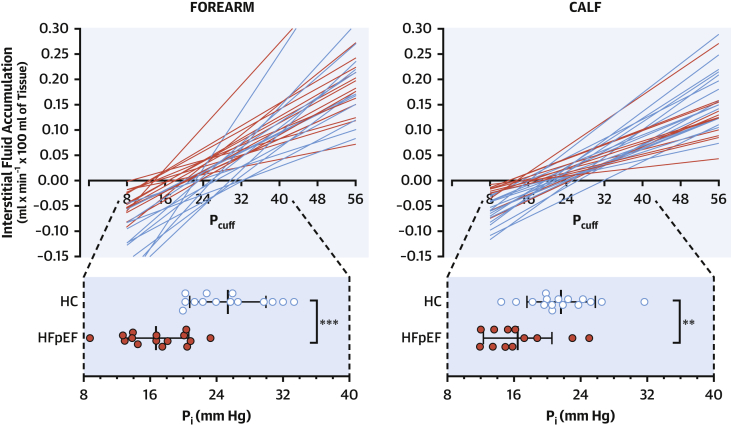
Microvascular Fluid Dynamics Net accumulation of interstitial fluid at different hydraulic pressures, at which cuffs were inflated to oppose venous drainage in the forearm **(left)** and the calf **(right)**. Each regression line corresponds to a participant. The slope of the lines is the microvascular coefficient of filtration (see Table 2). **Red lines** (HFpEF) intersect the x-axis at lower pressures compared with **blue lines** (HC subjects; scale magnification below): the intersect indicates the threshold above which interstitial fluid accumulation starts to develop (isovolumetric pressure [P_i_]). Data presented as mean ± SD; ∗∗p < 0.01, ∗∗∗p < 0.001. P_cuff_ = pressure applied to the cuff; other abbreviations as in Figure 1.

Because of motion artifacts, 3 forearm (1 patient with HFpEF, 2 HC subjects) and 4 calf (3 patient with HFpEF, 1 HC subject) tracings were excluded from the analysis of microfiltration; 1 additional calf tracing from a patient with HFpEF was excluded as per procedure-specific exclusion criteria (overt bilateral venous insufficiency). The net capillary fluid extravasation toward the interstitium (ml × 100 ml of tissue^–1^ × min^–1^), induced with progressively higher P_V_ by stepped inflations of proximal cuffs, was not increased in patients with HFpEF above HC subjects as was initially predicted. In fact, the slope of the regression line between interstitial fluid accumulation and cuff pressure was lower in patients with HFpEF compared with HC subjects in the calf (K_f_: 3.30 [IQR: 2.33 to 3.88] μl × 100 ml of tissue^–1^ × min^–1^ × mm Hg^–1^ vs. 4.66 [IQR: 3.70 to 6.15] μl × 100 ml of tissue^–1^ × min^–1^ × mm Hg^–1^; p = 0.010); a similar nonsignificant trend was observed in the forearm (5.16 [IQR: 3.86 to 5.43] μl × 100 ml of tissue^–1^ × min^–1^ × mm Hg^–1^ vs. 5.66 [IQR: 4.69 to 8.38] μl × 100 ml of tissue^–1^ × min^–1^ × mm Hg^–1^) (Table 2, Figure 3). However, patients with HFpEF also showed a reduced P_i_ (calf: 16 ± 4 mm Hg vs. 22 ± 4 mm Hg; p = 0.003; forearm: 17 ± 4 mm Hg vs. 25 ± 5 mm Hg; p < 0.001): in our in vivo experimental setting, this reflects the critical pressure up to which lymphatic drainage can fully compensate for the continuous extravasation of fluid at even low P_V_, and no net interstitial fluid accumulation occurs (i.e., the intercept of the lines with the x-axis) (Figure 3).

In summary, the clearance of interstitial fluid fails early to meet demands imposed by the capillary extravasation induced by hydraulic (venous) pressure in HFpEF.

## Discussion

For the first time in the understanding of HFpEF, our HAPPIFY study extended the concept of microvascular dysfunction to the lymphatic system. Despite absence of tissue Na^+^ hypertonic excess to facilitate edema, we showed that the lymphatic vessels of patients with HFpEF exhibit structural and molecular alterations in the skin and cannot effectively compensate for fluid extravasation and interstitial accumulation by commensurate drainage. Along with direct ex vivo evidence of primary dysfunctional small subcutaneous resistance arteries and rarefaction of skin blood vessels, as already described in myocardium (9) and in skeletal muscle (11,13), this functional and structural vascular impairment further supports the systemic nature of HFpEF syndrome (13).

### Rarefaction of microvessels

Impaired vasodilatation is an established characteristic of patients with HFpEF and contributes to exercise disability (4). In our study, this was confirmed not only by classical indirect approaches like brachial flow-mediated dilatation and post-ischemic reactive hyperemia, but also by the first ex vivo analysis of vascular endothelium-dependent (via acetylcholine) and endothelium-independent (via sodium nitroprusside) relaxation of systemic resistance arteries (Supplemental Figures 3 and 4). In vivo, however, any hyperemic flow response is critically affected also by the number of ultimate effectors (i.e., vessels), as demonstrated by experimental disruption of coronary microvasculature in pigs (30). In patients with HFpEF, the reduced coronary microvascular density found in autopsy samples (9) is likely to cause pathological heterogeneity and mismatch in blood supply and demand (31), thus impairing myocardial O_2_ extraction and performance. Similarly, exercise capacity in HFpEF is further reduced by low peripheral O_2_ extraction and skeletal muscle capillary density (10, 11, 12). Here, on the one hand, we showed that rarefaction of blood microvessels in HFpEF occurs also in the skin, by far the largest human organ and key in the regulation of body hemodynamics. On the other hand, and at variance with the previously mentioned largely “arteriocentric” paradigms, our findings prompt reconsideration of the role of microvasculature as a whole, to include functions other than vascular resistance homeostasis and structures other than arteries in HFpEF pathogenesis.

### Interstitium, microvasculature, and congestion in HFpEF

Congestion (i.e., edema) is central to HF syndrome (5), but to the best of our knowledge this study is the first to investigate the fine capillary–interstitium fluid homeostasis in HFpEF.

Over the last decade, excess Na^+^ has been reported in the skin and skeletal muscle of aged hypertensive patients (16), diabetic patients (17), and chronic kidney disease patients (18). This phenomenon had originally been described in rodents as water-independent (14) and capable of stimulating a lymphangiogenic signaling pathway to increase the drainage of tissue Na^+^ itself (15). Although a direct demonstration in HFpEF was missing, all of these are typical comorbidities of patients with the syndrome, and we hypothesized that hypertonic Na^+^ accumulation could drag excess fluid out of vessels and thereby favor edema. Although the concentration of Na^+^ in the skin of patients with HFpEF was high when contrasted to a previously reported cohort of young healthy subjects (20) (data recalled in Supplemental Tables 3 and 4 for reference), it was accompanied also by higher water content and was not different from control subjects of similar age distribution, likely reflecting the lack of overt decompensation of HF and edema in a nondependent site in our cohort of patients with stable HFpEF. The excess Na^+^ and water content in the skin of our participants compared with young healthy subjects is reminiscent of the age-dependent isotonic Na^+^ retention observed also in uncomplicated hypertensive patients, reflecting the expansion of the tissue extracellular volume (20); as in that cohort, the identical (Na^+^ + K^+^) concentration between study groups rules out any hypertonic Na^+^ excess. Accordingly, and contrarily to our initial expectations, the rate of tissue fluid accumulation induced by increasing hydraulic pressures (i.e., K_f_) was not higher in patients with HFpEF compared with HC subjects. In keeping with the dependence of K_f_ on the vascular surface available for fluid exchange and with the rarefaction of blood vessels observed in HFpEF, it actually was lower. It is possible that blood flow in HFpEF, similar to HC subjects when normalized by limb volume, was partially diverted from exchanging capillaries to functional shunting (31); in other conditions characterized by increase in local blood flow (e.g., postural tachycardia syndrome), K_f_ was elevated (24).

At variance with ex vivo preparations, our in vivo plethysmographic approach accounted not only for the capillary fluid extravasation regulated by Starling-Landis balance, but also for the simultaneous fluid drainage out of the tissue. In fact, contemporary direct measurements of the hydraulic and oncotic interstitial forces revealed that under most conditions there is no sustained reabsorption of interstitial fluid at the venous end of the capillary beds, that transvascular flow is generally unidirectional (from the capillary lumen to the interstitium), and that drainage, up to 8 l/day, is provided by lymphatics (32). Under the assumption that the osmotic pressure of albumin (σΠ) was similar between study groups based on similar plasma concentrations, the equilibrium hydraulic pressure above which net extravasation of fluids starts to overfill the interstitium (i.e., generate edema) is mostly determined by lymphatic drainage. Our results showed that this pressure (P_i_) was lower in patients with HFpEF compared with HC subjects in both arms and legs. A lower P_i_ and a higher CVP (as one can estimate by P_V_ values in Table 2) leaves patients with HFpEF with a much lower range of pressures that can be compensated for by their lymphatics. In other words, they appear to have reduced “lymphatic reserve” (Central Illustration).

**Central Illustration:**
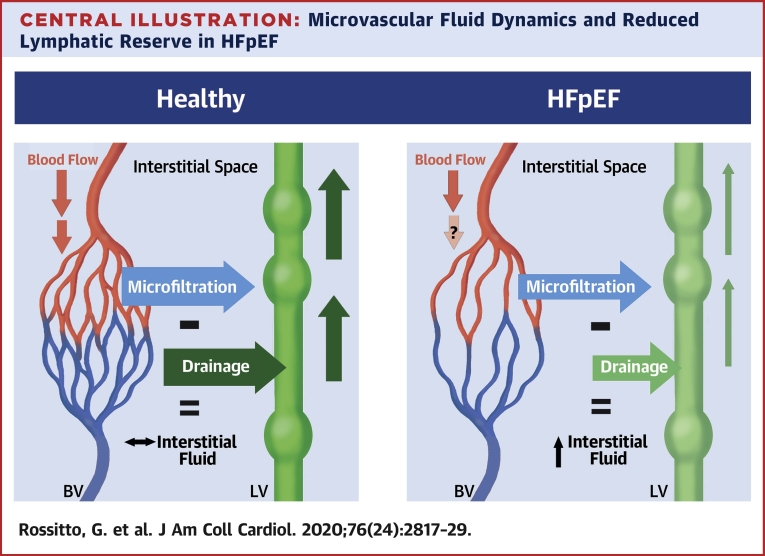
Microvascular Fluid Dynamics and Reduced Lymphatic Reserve in HFpEF In healthy subjects, the fluid filtering out of the capillary bed of the blood vasculature (BV) is evenly balanced by commensurate fluid drainage by lymphatic vasculature (LV); as a result, the physiological amount of interstitial fluid is homeostatically preserved. In heart failure with preserved ejection fraction (HFpEF), the net fluid extravasation tends to be lower because of the reduced vascular surface available for fluid exchange (i.e., capillary rarefaction) and the possible diversion of blood flow toward arteriovenous shunts; however, drainage by the impaired lymphatic system is inadequate to meet demands and facilitates accumulation of interstitial fluid at lower venous pressures. Both anatomical and functional defects **(light green)** could explain the reduced lymphatic reserve.

### The lymphatic system

Lymphatic vessels are organized into a series of coordinated functional units, each called lymphangion, separated by intraluminal 1-way valves and capable of spontaneous pumping activity. Their physiology has striking similarities with heart function, namely a systole-diastole cycle and a contractility modulated by pre- and afterload, conferring remarkable plasticity in response to increased requirements (33). The complexity of contractile machinery, valvular function, and electromechanical coordination suggests that also functional impairment, rather than the sole anatomical severance traditionally implicated in most secondary lymphatic disorders, could contribute to disease whenever an imbalance develops between microvascular filtration and lymphatic drainage. It has indeed been suggested that all “chronic edema could be considered as synonymous with lymphedema” (34), intended as relative lymphatic failure or inadequate reserve. Our functional results support this contention in HFpEF.

The morphological changes observed in skin lymphatics, with rarefaction of terminal capillaries and larger residual vessels, as well as the reduced gene expression of factors important for lymphangiogenesis and valve and vascular integrity maintenance (27,28), offer only preliminary clues as to what sustains the functional defect observed in HFpEF. In particular, we cannot conclusively identify cause and effect. Based on our results, we can only speculate as to whether a constitutive defect or a limited functional reserve of the lymphatic system is a primary predisposing factor toward an overt clinical syndrome when additional insults occur (i.e., comorbidity-driven chronic inflammation or elevated CVP) or, vice versa, a result of their long-term, potentially additive detrimental effects (35,36). The latter (i.e., a consequence of comorbidities and of target organ damage with diastolic dysfunction) would be in keeping with morphological changes and impaired maximal lymphatic pumping capacity reported in patients with a Fontan circulation, in whom chronically increased CVP would induce initial compensation of the afterload but long-term failure (26). Conversely, an underlying predisposing defect would remind of cases with secondary lymphedema, in which systemic alterations in lymphatic drainage precede the clinical onset of lymphedema and affect also the contralateral limbs (37,38). Detection of lymphatic impairment also in the upper limbs, in which not even trace edema was detectable in either group compared with the calves or ankles of some of our patients with HFpEF, suggests a systemic nature of the phenomenon, although involvement of other organ-specific microvascular beds remains to be investigated.

Regardless of ‘which comes first’ considerations, reduced capacity of the lymphatic system to prevent or minimize development of edema could mark the clinical and prognostic distinction between patients with uncomplicated comorbidity and those presenting as HFpEF (3). Moreover, by targeting edema and its dysfunctional and inflammatory implications, improving lymphatic function could improve quality of life, muscle performance, and exercise tolerance; disrupt vicious inflammatory cycles; and reduce the need or dose of diuretic treatment to maintain sustained decongestion. Of note, although routinely impractical in its surgical approach, successful external thoracic duct drainage in advanced HF has already shown therapeutic promise as a proof of concept (39).

### Study limitations

In addition to the aforementioned lack of causality demonstration, which warrants additional dedicated studies, other limitations deserve to be mentioned. Healthy subjects were chosen as control subjects and comparison of HFpEF with comorbidity-matched patients is missing; similarly, a comparison between patients with HFpEF with and without obesity (35) would further define the specificity of our findings. Of note, none of the drug classes in use in the study, including calcium-channel blockers (40), have been reported to adversely affect lymphatic function in vivo. For our conclusions, we assumed similar osmotic pressures of albumin (σΠ), critical for isovolumetric pressure ex vivo (32), between groups; as the accurate measurement of leakiness of blood vessels to albumin (i.e., σ) is cumbersome and unfeasible in clinical settings, the assumption was based on similar plasma albumin concentrations. Notably, even if excess permeability of blood vessels in HFpEF was demonstrated, the relative compensation provided by lymphatics would still remain inadequate. Finally, we assessed multiple physiological parameters, and we cannot exclude the presence of type I error; similarly, our sample size prevented subphenotyping of the HFpEF cohort.

## Conclusions

This study provides the first description of morphological and functional changes in the lymphatic vasculature in HFpEF, resulting in reduced clearance of extravasated fluid. Along with demonstration of a reduced blood microvascular density in the skin, such findings draw attention to a globally and systemically defective microcirculation. This definition extends beyond the traditional arterial dysfunction and calls for better understanding of the role of arteries, veins, lymphatics, and their mutual crosstalk for tissue fluid (and likely inflammatory) homeostasis in the pathogenesis of this multifaceted, syndromic disease.

Perspectives **COMPETENCY IN MEDICAL KNOWLEDGE:** In patients with HFpEF, systemic microvascular dysfunction, including vascular rarefaction, involves both capillary and lymphatic vessels, reducing lymphatic reserve and dysregulating vascular–interstitial fluid homeostasis.

**TRANSLATIONAL OUTLOOK:** Better understanding of the structural and molecular mechanisms responsible for reduced lymphatic reserve may facilitate the development of pharmacological interventions that improve interstitial fluid drainage in patients with HFpEF fluid drainage.

## Author Disclosures

This study was supported by British Heart Foundation Centre of Research Excellence Awards to Drs. Touyz, Delles, Petrie, and Rossitto (RE/13/5/30177 and RE/18/6/34217+). Dr. Touyz is supported by a British Heart Foundation Chair award (CH/12/29762). The authors have reported that they have no relationships relevant to the contents of this paper to disclose.
