# Supplementary material for: Reduced Lymphatic Reserve in Heart Failure With Preserved Ejection Fraction
Source: J Am Coll Cardiol. 2020 Dec 15;76(24):2817–29. doi: 10.1016/j.jacc.2020.10.022 (PMC7724570; doi:10.1016/j.jacc.2020.10.022)
Supplement: Supplemental Data [file mmc1.pdf]

Reduced Lymphatic Reserve in Heart Failure with Preserved Ejection Fraction

G. Rossitto[1], S. Mary[1], C. McAllister[3], K.B. Neves[1], L. Haddow[1], P. Rocchiccioli[2], N. Lang[1], C. Murphy[4], R.M. Touyz[1], M.C. Petrie[1,2], C. Delles[1]

SUPPLEMENTARY MATERIAL

Expanded methods.

Supplementary Table 1. ***Echocardiographic parameters.***

Supplementary Table 2. ***Macrovascular function.***

Supplementary Table 3. ***Characteristics of young healthy subjects, for contrast.***

Supplementary Table 4. ***Skin chemical analysis, contrasted with young healthy subjects.***

Supplementary Figure 1. ***Immunohistochemical analysis.***

Supplementary Figure 2. ***Plethysmography analysis.***

Supplementary Figure 3. ***Peripheral arterial function assessed in vivo.***

Supplementary Figure 4. ***Peripheral arterial function assessed ex vivo.***

Supplementary Figure 5. ***Association of dermal fat with water and Na^+ content.***

Supplementary references.

EXPANDED METHODS

Study protocol and subjects

Subjects with HFpEF identified from outpatient HF clinics in Glasgow, UK, and healthy volunteers with no history of cardiovascular or renal disease, hypertension or diabetes, recruited by public advertisement, were invited to an eligibility visit where inclusion and exclusion criteria were reviewed. HFpEF diagnosis was per the 2016 European Society of Cardiology guideline definition: (i) signs and/or symptoms of HF clinically stable for > 1 month, (ii) elevated BNP > 35 pg/ml, (iii) an ejection fraction ≥ 50% and (iv) evidence of structural heart disease (left atrial enlargement and/or left ventricular hypertrophy) or diastolic dysfunction.(1) Patients with history of recent (<3 months) cerebrovascular event, myocardial infarction or coronary revascularisation; significant valve disease; unstable coronary artery disease; hypertrophic/infiltrative cardiomyopathy or constrictive pericarditis; chronic kidney disease > stage 3 were not considered for participation. General exclusion criteria also entailed history of idiopathic oedema/capillary leak syndrome, myxedema, lymphatic obstruction; evidence of systemic inflammation at the time of study visit; active malignancy or any major hypercoagulable state or history of venous thrombosis/embolism on no ongoing anticoagulation; incapacity. Study procedure-specific exclusion criteria are listed below in the pertinent subsections. Between August 2017 and December 2018, 16 subjects with HFpEF and 16 healthy controls (HC) were recruited.

The eligibility and main visits were conducted in the morning, in a temperature-controlled room, asking participants to refrain from caffeine and smoking, to fast for >8 h (eligibility) or >4h from a light breakfast if prolonged fasting for the whole night and morning was deemed unfeasible/unsafe (main) and to avoid vasoactive (ACE inhibitors/ARBs, beta blockers, calcium channel blockers, nitrates) or diuretic medications on the morning of the visit.

The study procedures included history/physical examination; full blood count, BNP, HbA1c and renal, liver and thyroid function; urinary albumin-to creatinine ratio (morning spot sample); simultaneous bilateral brachial and calf blood pressure measurement; two-dimensional echocardiography; flow-mediated dilatation; pulse wave velocity; venous occlusion strain gauge plethysmography and a gluteal skin punch biopsy. Participants without specific contraindication (e.g. ongoing anticoagulation) were offered to participate in an optional sub-study involving surgical collection of extra skin and subcutaneous fat tissue around the site of the punch biopsy for dissection of resistance arteries and/or molecular biology.

Our study conforms to the Declaration of Helsinki and the protocol was approved by the West of Scotland Research Ethics Committee 3 (ref. 17/WS/0091) and Greater Glasgow and Clyde (GG&C) NHS Research and Development (ref. GN17CA152).

Blood pressure measurement

Participants underwent 3 consecutive bilateral simultaneous measurements of brachial artery blood pressure (BP) values with a calibrated oscillometric monitor after 5 minutes of supine rest; office BP was determined as the average of the 2nd and 3rd readings. Similar measurements were later performed on the calves, up to 3 times if tolerated. Mean blood pressure (MBP) was calculated as (0.33 × Systolic BP) + (0.67 × Diastolic BP); a difference > 3 mmHg in limb MBP between sides was accounted for in the calculation of arterial resistance to flow with plethysmography (see below).

Normotension as inclusion criterion for healthy controls was defined upon evidence of office BP < 140/90 mmHg, or confirmation by 24h ambulatory blood pressure monitoring for those who showed office BP values above such threshold, without any antihypertensive medication.

Pulse wave velocity (PWV)

Pulse wave velocity (PWV) measurements were performed with a SphygmoCor XCEL System, in a supine position after 5 minutes of rest. The carotid-femoral distance was calculated by the SphygmoCor software by subtraction method from the sternal notch to the top edge of the femoral (thigh) cuff and from the patient's carotid measurement site to the sternal notch. Once the system detected a carotid pulse signal of valid quality from the tonometer, a femoral cuff was inflated and the PWV measurement started automatically. All measurements were taken consecutively in duplicate, whenever possible, and averaged. Inaccurate measurements, according to the system automatic quality control, were discarded; in 3 study subjects (2 HFpEF, 1 control) no valid measurements could be obtained.

Echocardiography

Two-dimensional echocardiography with Doppler and tissue Doppler imaging was performed by a single operator during the eligibility visit, using a Siemens ACUSON SC2000 ultrasound system. Cardiac structure and function were blindly quantified off-line at the end of the study, as recommended by the American Society of Echocardiography/European Association of Cardiovascular Imaging.(2,3)

Brachial artery flow mediated dilatation (FMD)

FMD measurements were performed according to guidelines,(4) during the eligibility visit, with a semi-automated device (UNEX EF, Nagoya, Japan; https://unex.co.jp/ENG/unexef.htm). The device is equipped with an automated edge-detection software and a vessel tracking system which facilitates and continuously micro-correct probe positioning for steady images at the beginning and during the test. The H-shaped probe, generating one long-axis and two short-axis B-mode images, operates at ultrasound wave frequency of 8MHz. Blood velocity is simultaneously measured by the same

probe, with an automatically adapted flow sampling rate (7.1kHz, 5.9kHz, 5.0kHz, 4.0 kHz) and a sample volume adjusted to the internal vessel diameter. Intensity weighted mean velocity assuming a circular cross-section of the vessel and its time-average during the cardiac cycle are automatically calculated. Due to the dependence of velocity assessment on the angle of Doppler insonation and its fixed value in the UNEX EF device (75°), preventing absolute comparisons with different machines, averaged blood velocity (V) and the derived flow ($V \times \pi$ (vessel diameter/2)$^2 \times 60$) and shear rate ($8 \times V$/diameter) changes are reported as relative to baseline.

After BP measurement and 10 additional minutes of quiet supine rest, an occlusion cuff was placed around the right forearm, the ultrasound probe 5-10 cm proximal to the elbow and ECG leads on the wrists. Following measurement of the rest diameter (intima-intima) and blood velocity for at least 30 s, the cuff was inflated 50mmHg above systolic BP to induce forearm ischemia for 5 minutes. One minute before cuff release the tracking system automatically tracked the artery and adjusted the probe position to correct for small involuntary arm displacements; after deflation the vessel diameter was measured for 2 minutes, for a total of 3 minutes of ECG-triggered images. Scans judged as non-diagnostic at the time of the eligibility visit (if intima-intima was not stably visible throughout the reading, in case of gross arm misplacements and/or overt failures of the probe position adjusting software) were repeated at the beginning of the main visit under the same conditions. All the images and automated outputs were manually reviewed and analyzed with UNEX software off-line at the end of the study;(5) the best position for edge-to-edge diameter measurement was identified on the vessel and dynamically tracked by the automatic software throughout the course of the exam. FMD was calculated as [(Max Diameter – Rest Diameter)/Rest Diameter] × 100. Outputs from any scan previously judged as non-diagnostic but rescued by off-line analysis were averaged with results from the second scan, if available; persistently

non-diagnostic scans were excluded (1 HFpEF). The researcher who performed the analysis was blind to the group allocation of scans.

Surgical skin samples

All participants (except for 1 patient, due ineffective topical anesthesia) underwent a 4-mm skin punch biopsy on a gluteal external upper quadrant, after topical anesthesia with Na^{+}-free lidocaine cream (LMX4, Ferndale) as previously described.(6)

Surgical-specific exclusion criteria were allergy to lidocaine or chlorhexidine gluconate; chronic diffuse skin condition without uninvolved areas suitable for biopsy; history of keloid scar formation; known diagnosis of hepatitis B or C or HIV or decline to undergo a skin/gluteal biopsy. The excised skin sample was cut on the bench into two hemicylinders, each including both epidermis and dermis: one was fixed in PFA 2% for 8-10h at room temperature, washed in PBS and stored in 70% ethanol at 4°C until paraffin inclusion for subsequent histological analyses; the second was immediately put into a pre-cooled Eppendorf tube, frozen in dry ice and stored at -80°C until tissue chemical analysis. Participants who were eligible and consenting also to *ex vivo* sub-study, subsequently underwent a skin and subcutaneous fat biopsy (measuring approximately 2.5 × 1 × 1.5 cm) on the same gluteal site, upon anesthesia with subcutaneously-injected lidocaine 2%. Predefined additional exclusion criteria for this procedure included ongoing treatment with warfarin or other anticoagulant medication or severe obstructive iliac artery disease. A small portion of the excised dermis and epidermis was immediately frozen in dry ice and stored at -80°C for molecular biology; the remaining available tissue was used to dissect small resistance arteries for *ex vivo* functional testing.

***Ex vivo* assessment of vascular function.**

The protocol for the functional assessment of resistance arteries has been previously reported.(6,7) Briefly, small arteries were dissected from fat/dermis and cut into 2-mm ring segments, which were mounted on isometric wire myographs (Danish Myo Technology [DMT], Denmark) filled with 5ml of physiological saline solution [PSS; 119.0 mM NaCl, 4.7 mM KCl, 1.2 mM $MgSO_4.7H2O$, 24.9 mM $NaHCO_3$, 1.2 mM KH_2PO_4, 2.5 mM $CaCl_2$ and 11.1 mM glucose] and continuously gassed with a mixture of 95% O2 and 5% CO2 while being maintained at a constant temperature of 37±0.5°C. Following 30 minutes of equilibration, baseline tension was normalized as per DMT recommendations and internal diameter was estimated as reported; vessels with an internal diameter ≥ 500 µm, traditionally identified as the maximum size of resistance arteries,(8,9) were excluded from further analysis. After normalization, the viability of arterial segments was assessed by the addition of KCl (62.5mmol/L). Endothelium-dependent and -independent relaxation was assessed by a dose-response to acetylcholine (Ach; 10^{-10} - 3×10^{-5} M) or the nitric oxide (NO)-donor sodium nitroprussiate (SNP; 10^{-10} - 10^{-5} M), respectively, following pre-constriction with the thromboxane agonist U46619 dose that produced 75% of the maximal contractile response. Out of 16 subjects with a biopsy for vascular function (n = 7 HFpEF and 9 HC), 3 HC participants were excluded because no viable vessels (n=2) or only vessels of unsuitable size (n=1) could be dissected; one case with HFpEF was excluded because of spontaneous vasomotor activity generating early loss of vascular tone after pre-constriction, at doses of Ach or SNP unable to physiological induce relaxation in either group and automatically identified as outliers (Log EC_{50} Ach: -9.00; ROUT method, Q = 1%), possibly due to vessel damage in the mounting process.

Skin histochemical analysis

Our protocol for histochemical analysis has been extensively described elsewhere.(6) Briefly, frozen skin samples were macroscopically transversally cut into a superficial layer (including the epidermis and the immediately adjacent superficial dermis; ESD) and a deeper dermis layer (DD; Figure 3C) in a cold room, to prevent evaporation of moisture. Tissue water content was estimated by a gravimetric approach as wet weight (WW) – dry weight (DW), obtained after desiccation at 65°C for > 40 hours and assessed on a 5 decimal (0.00001 g) scale (Ohaus, DV214CD). Tissue Na^+ and K^+ were measured in the HNO_3-digested samples by flame photometry (Sherwood scientific, 410C) and expressed as absolute content (mmol/gDW) or concentration (mmol/l, after normalization by tissue water). During flame photometry analysis, operators were blind to group allocation of samples.

Skin microvascular immunofluorescence and image analysis

Paraffin embedded skin sections (5-µm-thick) including both epidermis and dermis were mounted on Superfrost Plus Adhesion slides (Fisher Scientific UK Ltd), deparaffinized in xylene and rehydrated through baths of ethanol solutions (100% to 50%) to dH2O. Sections from all participants were further processed in a single batch to maximize homogeneity of handling and staining. Antigen retrieval was performed by overnight incubation in Unitrieve (Innovex Biosciences) at 42°C. After blocking in 15% donkey serum (D9663, Sigma) + Tween20 0.05% in TBS for 1h at room temperature, sections were incubated overnight at 4°C with a goat anti-human Lyve-1 antibody (AF2089, R&D systems) at a concentration of 3 µg/ml in TBS/15% serum; matched isotype goat IgGs (Vector laboratories) were used as negative controls. On the following day, sections were washed and incubated with Alexa-fluor-488-conjugated donkey anti-goat IgG secondary antibody (2 µg/ml; Invitrogen) for 1h at room temperature and counterstained with Ulex europaeus agglutinin I (UEA-I) lectin conjugated to Rhodamine (RL-1062, Vector Laboratories), specifically binding glycoproteins

and glycolipids on human endothelial cells,(10) at a final concentration of 10 µg/ml in 1xTBS for 30 mins at RT. After treatment with Sudan Black 0.1% to minimize autofluorescence, slides were stained with DAPI (100 µg/ml; Life technologies), mounted in ProLong Gold anti-fade mounting media overnight at room temperature and then stored at 4°C in the dark. Tiled images of the entire epidermal line and the sub-papillary dermis (≥ 2 mm of minimal imaged thickness), where the blood and lymphatic network are consistently located (Supplemental figure 2), were acquired at 20× magnification using an inverted epifluorescence microscope (Axio Observer Z1, Zeiss) and a dedicated software (Zen Blue Program, Zeiss). Laser excitation and acquisition settings were maintained constant across all slides.

Automated image analysis for the quantification of microvessels was performed with ImageJ, with the operator blind to group allocation of samples. The 600-µm-thick dermal area starting from the epidermis-dermis junction and spanning the entire length of the biopsy, with the exception of portions affected by gross mounting/staining artefacts, was manually identified and selected (Supplemental figure 2). After splitting RBG channels, a standardized threshold algorithm was used for vessel detection and measurement of total Lyve1^{+} and lectin^{+} area (as % of total selected dermal area); the stain intensity threshold was set to exclude nonspecific staining, including the faint and inconsistent lectin^{+} staining of lymphatic endothelium (Supplemental figure 1). On thresholded images, microvascular density (n/tissue mm^2) and size (µm^2) were measured with the operator-independent '*analyze particles*' command, without circularity constraints due to variability in the orientation of dermal microvessels.

Dermal fat quantification was performed with ImageJ, after staining of additional sections from the same samples with Picrosirius Red for 1h. Adypocytes-occupied area was manually identified and expressed as % of total area for comparisons.

Skin gene expression analysis

Total RNA was extracted from the frozen skin samples from gluteal surgical biopsies (sub-study; n=17, 7 HFpEF and 10 HC) using QIAzol lysis reagent and RNAesy mini-column kit (Qiagen) according to the manufacturer's instructions. cDNA was generated from total RNA using the High-Capacity cDNA Reverse Transcription Kits (Applied Biosystems). After preamplification (TaqMan® PreAmp technology, Applied Biosystems) of target cDNA, a custom-made TaqMan® Array Card was used to perform quantitative gene expression of markers or growth/transcription factors specific to blood and/or lymphatic vessels, including: VEGF-A (Hs00900055_m1), VEGF-B (Hs00173634_m1), VEGFR1 (or FLT1; Hs01052961_m1), VEGFR2 (or KDR; Hs00911700_m1), VE-cadherin (Hs00901465_m1), Lyve-1 (Hs00272659_m1), Podoplanin (Hs00366766_m1), Prox-1 (Hs00896293_m1), VEGF-C (Hs01099203_m1), VEGFR3 (Hs01047677_m1). Real-time PCR was run with samples in duplicate in a QuantStudio™ 12K Flex System. Gene expression levels were compared and presented as ΔCt values, with β-actin (Hs99999903_m1) as housekeeping gene based on evidence of CT consistency between study groups.

Venous occlusion strain gauge plethysmography and derived parameters

Strain gauge plethysmography (EC6, Hokanson) was used to measure forearm and calf arterial blood flow (Φ), venous pressure (P_V) and net fluid extravasation toward the interstitium at increasing venous occluding pressures, as described.(11-13) Clinical evidence of chronic venous insufficiency/post-thrombotic syndrome, lymphedema or history of dissection of lymph-nodes draining the limb were limb-specific exclusion criteria for segmental assessment on that side.

The plethysmograph was connected to a *PowerLab* data acquisition device (ADInstruments) and measured limb volume changes (*ml/100 ml of tissue*, or percentage) were recorded and later analyzed by *LabChart* software.

Patients were positioned almost supine, except for an approximately 10° tilt of the back, with their forearm and calf maintained at the height of the right atrium by in-house made supports. Inflatable cuffs for venous occlusion cuffs were placed proximal to the strain gauges. Measurements were performed in the non-dominant arm and ipsilateral calf, unless local contraindications/exclusion criteria (e.g. previous surgery or stroke, venous varices) applied. After a >15 min resting period and limb volume tracing stabilization, we determined P_V by gradually increasing the occlusion pressure until any volume change was detected. Under the experimental in use, with the limbs at the height of the right atrium, PV is a valid surrogate for central venous pressure (CVP).(14-16)

Blood flow measurements were performed in at least quintuplicate from the rate of change in limb volume after consecutive cycles of sudden venous occlusion to 45 mmHg (E20 Rapid Cuff Inflator, Hokanson). Forearm and calf BP were simultaneously measured on the contralateral limbs free of the gauges; for the calculation of arterial resistance to flow as $(MBP - P_V)/\Phi$ in the assessed limb ($mmHg/ml \times 100ml^{-1}$ per min), measured MAP was corrected by any > 3 mmHg side difference previously identified.

The methods used for the assessment of microvascular filtration parameters are evolutions of those originally used by Starling and Landis;(17,18) their rationale and technical details have been previously described.(11,12) Briefly, we used 8-mmHg occluding cumulative pressure steps, lasting 3.5 minutes each and starting at the first multiple exceeding P_V up to a maximum of 56 mmHg or less, if diastolic BP was lower, in order to avoid local sympathetic reflex mechanisms.(19) The pressure applied to the cuff (P_{cuff}) is transmitted to the veins and, when exceeding P_V, it induces a volume change due to venous filling. At lower steps, after a curvilinear initial phase lasting approximately 1 minute, it reaches a plateau (Suppl Figure 2A). However, above a certain equilibrium pressure up to which any fluid filtering across the microvascular interface due to Starling-Landis transcapillary forces is being removed at an

equivalent rate by lymphatic drainage,(20-22) the limb volume continues to increase linearly with time after completion of venous filling (Supplemental Figure 2B). This equilibrium pressure, above which lymphatics cannot compensate for filtration and the interstitium enlarges at a rate proportionate to P_{cuff}(20) is called isovolumetric pressure (P_i).

At high P_{cuff} steps, the time needed to and the proportion of limb volume change due to further venous distention decreases, with an increase in net fluid extravasation accumulating in the interstitium. The time course (slope) of this fluid accumulation at each pressure step was calculated off-line (*LabChart*) as the averaged first derivative from portions of tracing devoid of motion artifacts,(11) excluding the initial curvilinear phase of venous filling (fixed for all at: 90s at 8 mmHg, 80s at 16, 70s at 24, 60s at 32, 50s at 40, 40s at 48 and 30s at 56). Least-square fitting (Prism, GraphPad) of at least 3 valid points was used to identify the linear association of interstitial fluid accumulation with the occlusion pressure for each limb/participant, in order to determine the slope (microvascular filtration coefficient, K_f) and the intercept with the pressure axis (i.e. P_i, when the net increase in limb tissue volume is null, as filtration – lymphatic drainage = 0; Suppl Figure 1C). In case of less than 3 pressure steps with reliable values for linear regression, the limb tracing was excluded from analysis (arm: HFpEF = 1, controls n= 2; calf: HFpEF = 3, controls n= 1); one additional calf tracing from a patient with HFpEF had been excluded as per procedure-specific exclusion criteria (overt bilateral venous insufficiency). The tracing analysis was conducted blind to the group allocation of participants.

Statistical analysis

No previous data on K_f were available in HFpEF or similar aged populations for formal power calculation. Based on the heterogeneity of HFpEF and data from different diseases (POTS, type 1 diabetes), we estimated that a sample size of n = 20/group allowed sufficient power to detect the predicted difference in the coefficient of filtration (increase) and in

isovolumetric pressures (decrease) between patients with HFpEF and age/sex-matched controls, as well as differences in skin Na^+ based on pilot data in young healthy subjects.(6) After conclusion of the Ethics-approved duration of the study and recruitment of 16/20 subjects per group, an interim analysis was conducted. The results, herein presented, showed a trend for the coefficient of filtration opposite to what predicted and highly significant differences in isovolumetric pressure. Accordingly, recruitment was stopped for futility in relation to the primary hypothesis and no study extension was requested.

Statistical analysis was performed using Prism (version 8, GraphPad Software) and SPSS (version 25, IBM).

Categorical variables are presented as absolute numbers and percentages and compared by $\chi 2$ test. Continuous variables were tested for normality of distribution by graphical plot and Kolmogorov-Smirnov test; they are presented as mean ± SD (or SEM when stated) or median (interquartile range), as appropriate. Parametric (t-student) or non-parametric (Wilcoxon) unpaired unadjusted tests were used for comparison of primary (microvascular) and secondary endpoints between groups, accordingly. The combined effect of time and group on shear rate during FMD was tested by two-way ANOVA; time-clusters for differences between curves were defined based on ≥ 3 significant differences at consecutive timepoints based on multiple-t-test.

Least square fit was used for both non-linear regression (*ex vivo* vascular function; log(agonist) vs. response, no slope constraint) and linear regression (Na^+/water content in skin biopsies; interstitial fluid accumulation and calculations of K_f and P_i). Comparisons of curves for vascular function and skin Na^+/water regression slopes was performed by the extra sum-of-squares F test. Correlations were assessed by Spearman test.

The α level was set at 0.05 and all statistical tests were 2-tailed.

Supplementary Table 1. Echocardiographic characteristics.

Variables	HC (n=16)	HFpEF (n=16)	p
**Diastolic septal wall thickness** (cm)	**1.0 ± 0.2**	**1.3 ± 0.2**	**<0.001**
**Diastolic posterior wall thickness** (cm)	**1.0 ± 0.1**	**1.3 ± 0.2**	**<0.001**
**LV mass index** (g/m^2)	**84.3 ± 17.9**	**127.8 ± 26.6**	**<0.001**
**LV end-diastolic volume index** (ml/m^2)	41.3 ± 10.0	44.2 ± 6.9	0.367
**LV end-systolic volume index** (ml/m^2)	15.2 ± 4.1	17.0 ± 4.3	0.140
**LV ejection fraction** (%)	63.2 ± 3.5	60.8 ± 6.7	0.222
**LA volume index** (ml/m^2)	**23.3 ± 6.1**	**46.8 ± 12.8**	**<0.001**
**E velocity** (cm/s)	**68.1 ± 14.7**	**89.8 ± 25.6**	**0.006**
**A velocity** (cm/s; SR only)	78.3 ± 15.1	76.8 ± 29.8	0.865
**E/A ratio** (SR only)	0.9 ± 0.2	1.2 ± 0.5	0.198
**LV e' velocity – average** (cm/s)	8.6 ± 1.9	8.7 ± 1.6	0.900
**LV e' velocity – average** (cm/s; SR only)	**8.6 ± 1.9**	**7.3 ± 0.7**	**0.022**
**LV a' velocity – average** (cm/s; SR only)	**11.8 ± 2.0**	**8.7 ± 2.9**	**0.009**
**LV s' velocity – average** (cm/s)	8.1 ± 1.2	7.4 ± 1.2	0.142
**LV E/e' ratio – average**	**8.0 ± 1.6**	**10.8 ± 2.9**	**0.001**
**LV E/e' ratio – average** (SR only)	**8.0 ± 1.6**	**11.3 ± 3.2**	**0.025**
**TV regurgitation velocity** (m/s)	**2.1 ± 0.1**	**2.6 ± 0.5**	**0.005**

Data presented as n (%) for qualitative variables and mean ± SD for quantitative variables. LV = left ventricle. LA = left atrium. SR = sinus rhythm at the time of exam (n=8). LV tissue Doppler values are average of septal and lateral values. TV = tricuspid valve.

Supplementary table 2. Classical vascular function.

**Variables**	**HC (n=16)**	**HFpEF (n=16)**	**p**
**Carotid-femoral Pulse Wave Velocity** (m/s)	6.8 ± 1.1	8.6 ± 1.3	**0.001**
**Brachial artery Flow-Mediated Dilatation (FMD)**			
**Rest internal diameter** (mm)	3.78 ± 0.50	3.94 ± 0.67	0.438
**Maximal internal diameter** (mm)	3.99 ± 0.53	4.13 ± 0.70	0.538
**FMD** (mm)	**0.21 ± 0.06**	**0.15 ± 0.08**	**0.033**
**FMD** (% rest)	**5.6 ± 1.5**	**3.9 ± 2.1**	**0.014**
**Peak shear rate** (× rest)	**6.7 ± 1.7**	**4.6 ± 1.6**	**0.002**
**Time-to-peak shear rate** (sec)	**7.6 ± 2.8**	**10.9 ± 5.4**	**0.041**

Data presented as n (%) for qualitative variables and mean ± SD for quantitative variables.

Supplementary Table 3. Characteristics of young healthy subjects for contrast.

	SOWAS	*HAPPIFY*	
**Variables**	**Y-HC (n=17)**	*HC (n=16)*	*HFpEF (n=16)*
**Females**	**11 (64.7%)**	*11 (68.8%)*	*10 (62.5%)*
**Age** (years)	**25 ± 5**	*68 ± 5*	*72 ± 6*
**BMI** (kg/m^2)	**23.8 ± 2.9**	*25.1 ± 2.9*	*33.9 ± 4.4*
**BSA**	**1.80 ± 0.25**	*1.78 ± 0.21*	*2.00 ± 0.28*
**SBP** (mmHg)	**112 ± 9**	*130 ± 14*	*146 ± 21*
**DBP** (mmHg)	**64 ± 8**	*73 ± 8*	*71 ± 14*
**HR** (beats per minute)	**67 ± 10**	*60 ± 7*	*65 ± 16*
**Na^+** (mmol/l)	**140 (140-141)**	*140 (139-142)*	*140 (136-142)*
**K^+** (mmol/l)	**4.2 (4.1-4.5)**	*4.5 (4.2-4.7)*	*4.2 (4.0-4.7)*
**Urea** (mmol/l)	**4.8 ± 1.2**	*5.5 (4.7-6.0)*	*6.4 (5.9-9.8)*
**Creatinine** (umol/l)	**75 ± 13**	*71 (59-84)*	*79 (67-90)*
**eGFR** (CKD-EPI; mL/min/1.73 m^2)	**106 ± 16**	*81.7 ± 11.0*	*70.9 ± 17.0*
**urinary ACR** (mg/gCr)	**4.7 (2.2-10.5)**	*4.8 (3.2-6.1)*	*33.9 (10.7-87.4)*

Data from HAPPIFY participants are contrasted to results from a cohort of young healthy subjects (Y-HC) from a pilot study already reported elsewhere (SOWAS [SOdium and WAter Skin Balance - Insights into Local Regulation] study, approved by the University of Glasgow, MVLS College Ethics Committee [ref.200170153] and conducted between July and December 2018)(6). For females (n=11), data and samples were collected in the early follicular phase of their menstrual cycle, just after period termination. Due to excess male participants in the original SOWAS cohort (n=18) compared to HAPPIFY, sex-matching for the herein presented comparison was achieved by random selection of 6 (by attribution of a randomly generated number and selection of the lowest 6).

Data presented as n (%) for qualitative variables and mean ± SD or median (interquartile range), as appropriate, for quantitative variables. BMI = Body Mass Index. BSA = Body Surface Area (Mosteller). SBP = office systolic blood pressure, DBP = office diastolic blood pressure; u-ACR = urinary albumin to creatinine ratio (random urine).

Supplementary Table 4. Skin chemical analysis, contrasted with young healthy subjects.

	SOWAS	***HAPPIFY***	
**Variables**	Y-HC (n=17)	*HC (n=16)*	*HFpEF (n=16)*
**Epidermis / Superficial Dermis (ESD)**			
**Water content** (%WW)	70.6 ± 1.3	*72.0 ± 1.0*	*71.3 ± 1.4*
**Na^+ content** (µmol/gDW)	264 ± 18	*304 ± 21*	*296 ± 33*
**Na^+ concentration** (mmol/l)	109.9 ± 4.9	*118.1 ± 6.2*	*118.6 ± 7.4*
**K^+ content** (µmol/gDW)	99 ± 12	*90 ± 12*	*75 ± 10*
**K^+ concentration** (mmol/l)	41.0 ± 4.6	*34.7 ± 4.3*	*30.3 ± 4.0*
**Na^+ + K^+ concentration** (mmol/l)	150.9 ± 6.4	*152.7 ± 7.0*	*150.0 ± 6.2*
**Deep Dermis (DD)**			
**Water content** (%WW)	65.6 ± 3.8	*61.7 ± 4.6*	*56.0 ± 5.8*
**Na^+ content** (µmol/gDW)	228 ± 39	*206 ± 43*	*162 ± 47*
**Na^+ concentration** (mmol/l)	117.4 ± 7.2	*125.1 ± 8.3*	*125.2 ± 10.9*
**K^+ content** (µmol/gDW)	49 ± 13	*34 ± 8*	*22 ± 3*
**K^+ concentration** (mmol/l)	24.9 ± 4.2	*20.4 ± 2.9*	*17.2 ± 2.9*
**Na^+ + K^+ concentration** (mmol/l)	142.4 ± 7.3	*145.5 ± 9.5*	*142.1 ± 11.3*

Skin biopsy surgical approach, visit conditions, sample processing and analysis for the young healthy controls (Y-HC) from the SOWAS study (see supplemental table 3) were the same as described for HAPPIFY. Data presented as mean ± SD. WW = wet weight; DW = dry weight.

No statistics is deliberately computed between different studies; however, please note: 1) the increase in water and Na^+ content (in ESD) and Na^+ concentration (in both ESD and DD), as well as the decrease in K^+ content and concentration (in both ESD and DD), consistent with histological changes associated with ageing(6); 2) the decrease in DD water and Na^+ content across groups, consistent with subcutaneous fat distribution (and BMI as a surrogate measure; please see supplemental table 3); 3) the similar total cationic concentration across groups, suggesting consistent lack of any hypertonic Na^+ accumulation.

Supplementary figure 1. Immunohystochemistry, methods.

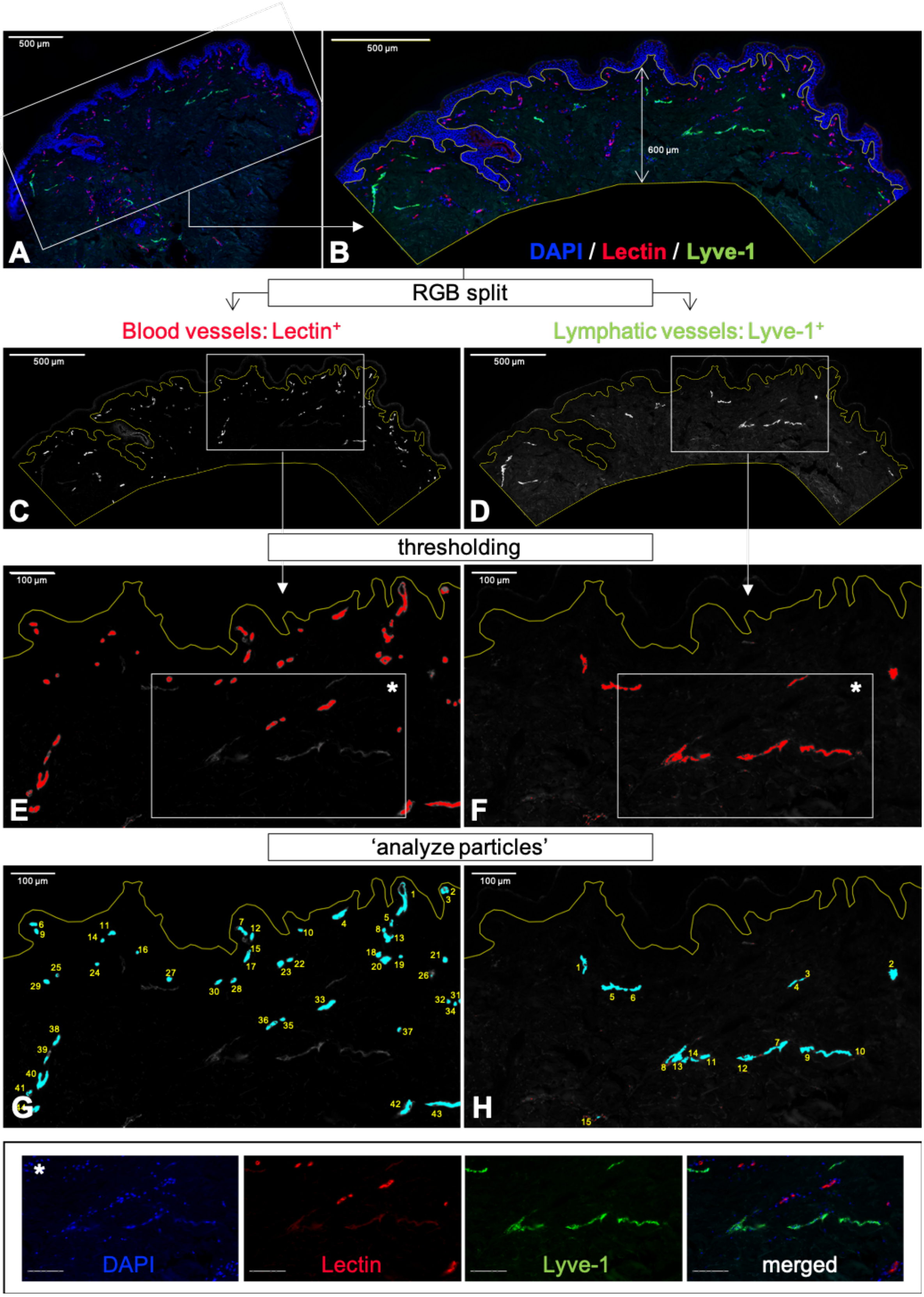

Panel A: Representative tiled image of the entire epidermal line (nuclei in blue; DAPI) and dermis. Dense blood (lectin$^+$, red) and lymphatic (lyve-1$^+$, green) vascular networks were located close to skin annexes and in the subpapillary dermis. *Panel B*: the 600-µm-thick dermal area starting from the epidermis-dermis junction was selected as the region of interest (ROI, in yellow). The red (*panel C, E, G*) and green (*panel D, F, H*) channels were split to characterize blood and lymphatic vascular network anatomy, respectively, by standardized thresholding (*E and F*) and 'analyze particles' (*G and H*, count and %area of total ROI). Insert (*) at bottom: the stain intensity threshold for lectin was set to exclude the low and inconsistent lectin$^+$ staining of lymphatic endothelium; scale bars = 100 µm.

Supplementary figure 2. Plethysmographic methods.

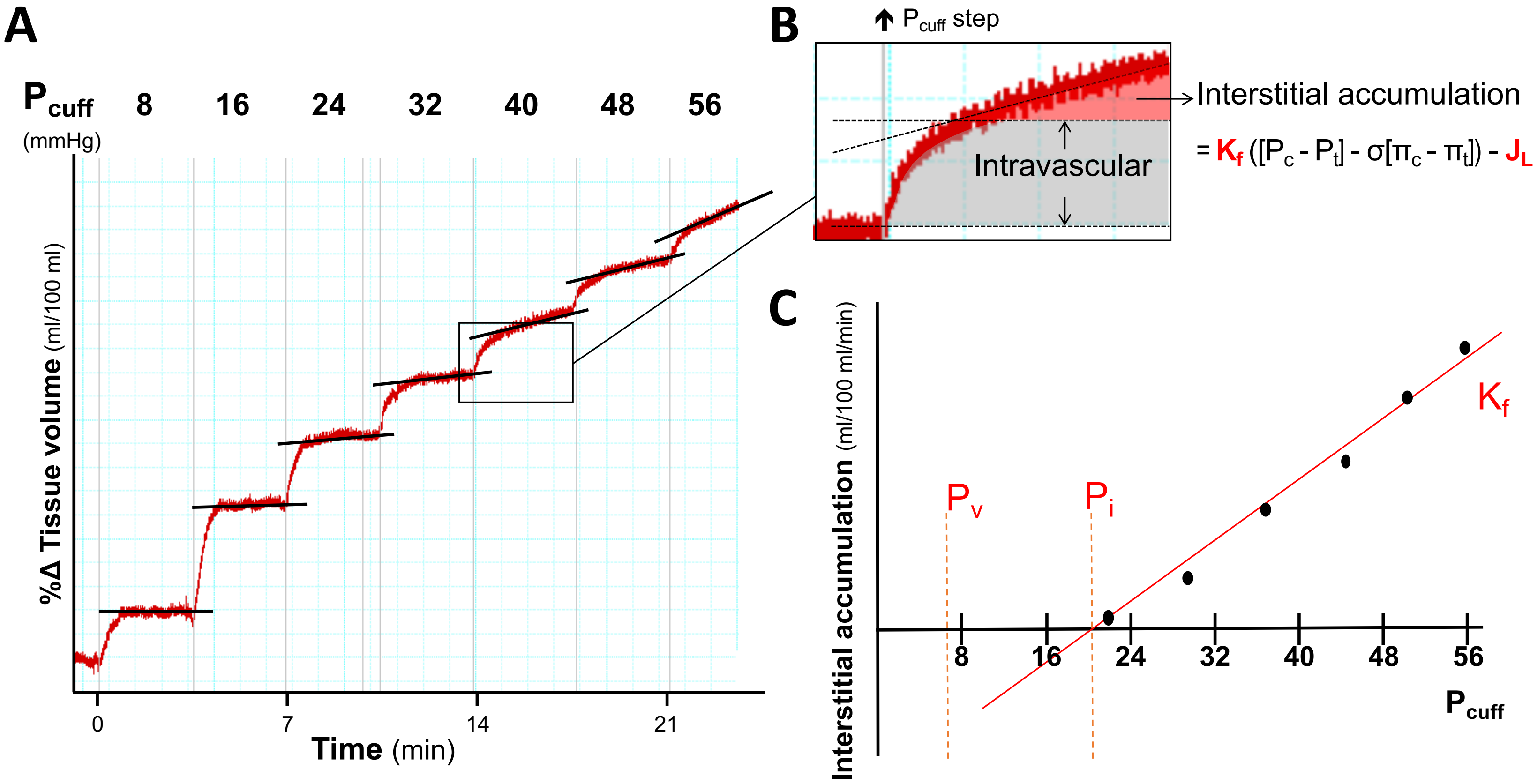

Panel A: Black lines showing increasingly steep changes in limb volume during our cumulative 8-mmHg pressure steps protocol are superimposed to a representative limb plethysmographic tracing (red). *Panel B*: inset from panel A, showing the intravascular (venous filling) and the interstitial component of limb volume changes after an increase in the cuff pressure, opposing venous drainage and backward increasing the hydraulic pressure at a capillary level. In our *in vivo* set up, net interstitial accumulation equals the difference between microvascular filtration (governed by Starling-Landis forces between capillary lumen [$_c$] and tissue [$_t$]) and lymphatic drainage (J_L). *Panel C*: interstitial fluid accumulation rates plotted against occluding (hydraulic) pressures. P_V = peripheral venous pressure; Pi = isovolumetric pressure, above which lymphatics cannot compensate for filtration and oedema develops; K_f = slope of the regression line, representing the coefficient of filtration in Starling-Landis equation. Please see (extended) methods for further details.

Supplementary figure 3. Peripheral arterial function assessed *in vivo*.

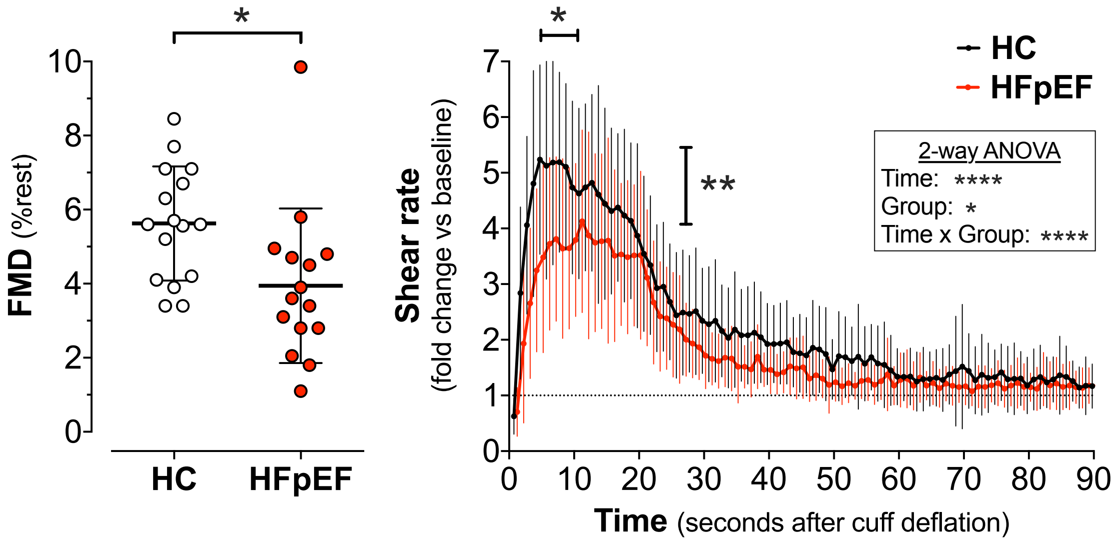

Left panel: endothelium-dependent brachial artery flow-mediated dilatation (FMD, expressed as percentage change from baseline) was reduced in patients with HFpEF (red) compared to healthy controls (HC, B/W). *Right panel:* shear rate imposed on the endothelial surface of the vascular wall during the post-ischemia hyperemic flow response (i.e. reactive hyperemia relative to baseline, the stimulus to FMD(23,24)) was also reduced HFpEF (red) compared to HC (black). Vertical and horizontal black capped lines identify differences in magnitude of and time to peak shear rate, respectively, between groups. Inset: influence of time, group and interaction on hyperemic response curve profile (2-way ANOVA). Please note the very early divergence between hyperemic responses, likely explained at least in part by microvascular rarefaction (please see Figure 3). All figure data are presented as mean ± SD; * p<0.05, ** p<0.01, **** p<0.0001.

Supplementary figure 4. Peripheral arterial function assessed *ex vivo*.

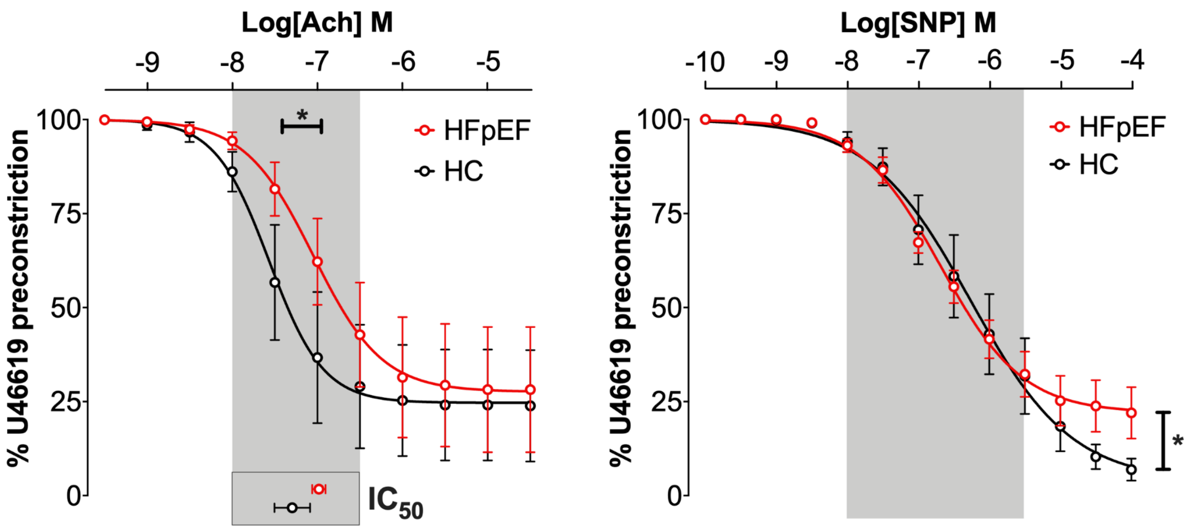

Left panel: endothelium-dependent relaxation to a cumulative concentration curve of acetylcholine (Ach), expressed as percentage of U46619-induced pre-constriction. Subcutaneous resistance arteries of patients with HFpEF showed reduced vasodilation, shown by the right-shift in the HFpEF curve compared to HC (Log of the IC_{50} (half maximal inhibitory concentration; inset) = -7.04 [95% CI: -7.07 to -7.02] M vs -7.57 [-7.60 to -7.53] M, $p < 0.001$): concentrations of Ach required for autoregulation of peripheral blood flow were ~5 times higher in HFpEF than controls. *Right panel*: endothelium-independent relaxation to a cumulative concentration curve of sodium nitroprusside (SNP), expressed as percentage of pre-constriction, revealing a lower maximal relaxation to SNP in HFpEF. Grey areas highlight the steepest portions of the curves (and relative Ach or SNP concentration ranges, accordingly), critical for fine-tuned peripheral modulation of arterial resistance and flow autoregulation. Horizontal and vertical and black capped lines identify differences in IC_{50} and maximal relaxation, respectively, between groups. All data are shown as mean ± SEM; n = 6/group; vessels from four additional subjects were excluded due to lack of viable vessels, unsuitable vessel size or excess vasomotion [n= 3 HC, 1 HFpEF], as detailed in the supplementary methods; * $p<0.05$

Supplementary figure 5. Association of dermal fat with water and Na^+ content.

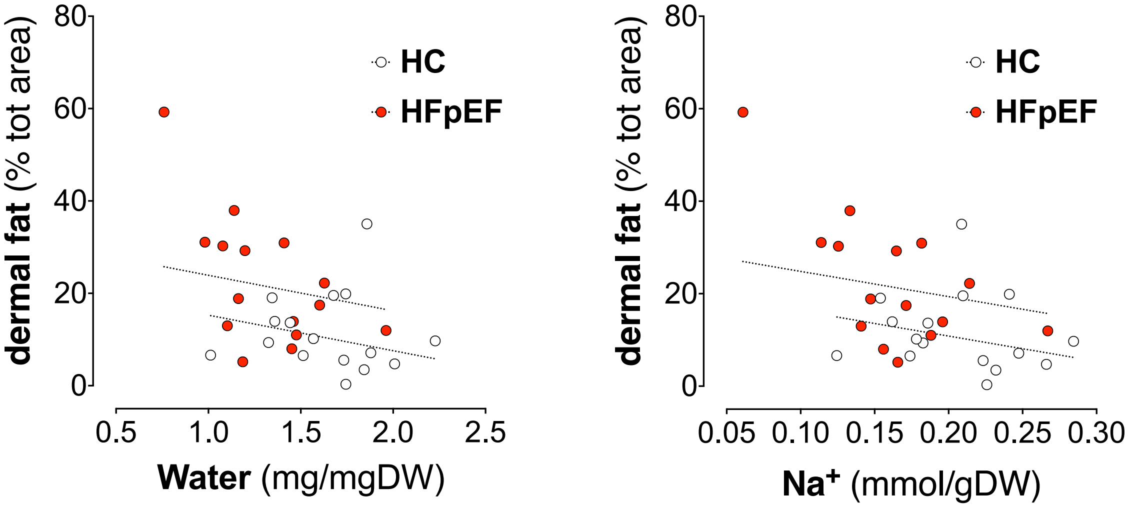

Both water and Na content in the dermis were inversely associated with dermal fat content, semi-quantitatively assessed by fat-occupied % of biopsy area in histology sections (Spearman $\rho = -0.415$, $p = 0.020$ and $\rho = -0.431$, $p = 0.016$, respectively). No differences were found in the slope of the associations at extra sum-of-squares F test between study groups.

SUPPLEMENTARY REFERENCES

1. Ponikowski P, Voors AA, Anker SD et al. 2016 ESC Guidelines for the diagnosis and treatment of acute and chronic heart failure: The Task Force for the diagnosis and treatment of acute and chronic heart failure of the European Society of Cardiology (ESC)Developed with the special contribution of the Heart Failure Association (HFA) of the ESC. Eur Heart J 2016;37:2129-200.
2. Lang RM, Badano LP, Mor-Avi V et al. Recommendations for cardiac chamber quantification by echocardiography in adults: an update from the American Society of Echocardiography and the European Association of Cardiovascular Imaging. J Am Soc Echocardiogr 2015;28:1-39.e14.
3. Nagueh SF, Smiseth OA, Appleton CP et al. Recommendations for the Evaluation of Left Ventricular Diastolic Function by Echocardiography: An Update from the American Society of Echocardiography and the European Association of Cardiovascular Imaging. J Am Soc Echocardiogr 2016;29:277-314.
4. Thijssen DH, Black MA, Pyke KE et al. Assessment of flow-mediated dilation in humans: a methodological and physiological guideline. Am J Physiol Heart Circ Physiol 2011;300:H2-12.
5. Dobbie LJ, Mackin ST, Hogarth K et al. Validation of semi-automated flow-mediated dilation measurement in healthy volunteers. Blood Press Monitor 2020.
6. Rossitto G, Mary S, Chen JY et al. Tissue sodium excess is not hypertonic and reflects extracellular volume expansion. Nat Commun 2020;11:4222.
7. Montezano AC, Lopes RA, Neves KB, Rios F, Touyz RM. Isolation and Culture of Vascular Smooth Muscle Cells from Small and Large Vessels. Methods in molecular biology (Clifton, NJ) 2017;1527:349-354.
8. Mulvany MJ, Aalkjaer C. Structure and function of small arteries. Physiol Rev 1990;70:921-61.
9. Heagerty AM, Aalkjaer C, Bund SJ, Korsgaard N, Mulvany MJ. Small artery structure in hypertension. Dual processes of remodeling and growth. Hypertension 1993;21:391-7.
10. Holthofer H, Virtanen I, Kariniemi AL, Hormia M, Linder E, Miettinen A. Ulex europaeus I lectin as a marker for vascular endothelium in human tissues. Laboratory investigation; a journal of technical methods and pathology 1982;47:60-6.
11. Gamble J, Gartside IB, Christ F. A reassessment of mercury in silastic strain gauge plethysmography for microvascular permeability assessment in man. J Physiol 1993;464:407-22.
12. Gamble J. Realisation of a technique for the non-invasive, clinical assessment of microvascular parameters in man; the KM factor. Eur Surg Res 2002;34:114-23.

13. Wilkinson IB, Webb DJ. Venous occlusion plethysmography in cardiovascular research: methodology and clinical applications. British J Clin Pharmacol 2001;52:631-46.

14. Amar D, Melendez JA, Zhang H, Dobres C, Leung DH, Padilla RE. Correlation of peripheral venous pressure and central venous pressure in surgical patients. J Cardiothorac Vasc Anesth 2001;15:40-3.

15. Tugrul M, Camci E, Pembeci K, Al-Darsani A, Telci L. Relationship between peripheral and central venous pressures in different patient positions, catheter sizes, and insertion sites. J Cardiothorac Vasc Anesth 2004;18:446-50.

16. Choi SJ, Gwak MS, Ko JS et al. Can peripheral venous pressure be an alternative to central venous pressure during right hepatectomy in living donors? Liver Transpl 2007;13:1414-21.

17. Starling EH. On the Absorption of Fluids from the Connective Tissue Spaces. J Physiol 1896;19:312-26.

18. Krogh A, Landis EM, Turner AH. The movement of fluid through the human capillary wall in relation to venous pressure and to the colloid osmotic pressure of the blood. J Clin Invest 1932;11:63-95.

19. Henriksen O. Local sympathetic reflex mechanism in regulation of blood flow in human subcutaneous adipose tissue. Acta physiologica Scandinavica Supplementum 1977;450:1-48.

20. Stewart JM. Microvascular filtration is increased in postural tachycardia syndrome. Circulation 2003;107:2816-22.

21. Michel CC. Microvascular permeability, venous stasis and oedema. International angiology 1989;8:9-13.

22. Bauer A, Christ F, Gamble J. Can lymphatic drainage be measured non-invasively in human limbs, using plethysmography? Clini Sci 2004;106:627-33.

23. Lee JF, Barrett-O'Keefe Z, Garten RS et al. Evidence of microvascular dysfunction in heart failure with preserved ejection fraction. Heart 2016;102:278-84.

24. Flammer AJ, Anderson T, Celermajer DS et al. The assessment of endothelial function: from research into clinical practice. Circulation 2012;126:753-67.
